# Characterization of humic acids from original coal and its oxidization production

**DOI:** 10.1038/s41598-021-94949-0

**Published:** 2021-07-28

**Authors:** Shuangdui Yan, Naiyu Zhang, Juan Li, Yanan Wang, Yue Liu, Mengyao Cao, Qiuyan Yan

**Affiliations:** 1 College of Resources and Environment, Shanxi Agricultural University, Taigu, 030801 Shanxi China; 2 Key Laboratory of Plant Nutrition and Fertilizer, Ministry of Agriculture and Rural Affairs/Institute of Agricultural Resources and Regional Planning, Chinese Academy of Agricultural Sciences, Beijing, 100081 China; 3 Institute of Wheat Research, Shanxi Agricultural University, Linfen, 041000 China

**Keywords:** Environmental impact, Environmental sciences

## Abstract

Five coal samples obtained from Chinese coal-producing areas were oxidized by hydrogen peroxide (H_2_O_2_), and humic acids (HAs) were derived from original coal and its oxidizition samples. HAs were characterized by physical and chemical methods, between which was also comparison. Yield, ash, aromaticity, molecular weight and functional group of HAs showed variance between original coals. While, yield, molecular weight, and the quantity of oxygen-containing groups of HAs increased more from coals oxidized with H_2_O_2_. However, the increase of oxygen-containing functional groups depended on original coals. For Yimin lignite, the oxidation of H_2_O_2_ could obviously improve the carboxyl group content of HAs, thus promoting the adsorption of nitrogen. This study demonstrated that oxidation of coal by using H_2_O_2_ was one pretreatment way to obtain and modify HAs which could be used as prerequisite and functional material in agricultural field.

## Introduction

Humic acids (HAs) is a mixture of natural amorphous colloid formed through various biological and abiotic degradation processes of animal and plant residues^1^, which are featured by complex molecular structure, chemical composition, chemical reactivity and decomposition resistance^2^. HAs widely exist in soil, coal and natural water body, and meanwhile their composition, structure and application have been widely studied in agriculture^3–5^. For example, HAs could increase soil organic matter, fertilizer effectiveness and improve the availability of phosphate in soil^6–8^. HAs mainly contain hydroxyl, carboxyl and quinone, which demonstrate excellent physiological activity, adsorption, exchange and other properties^9,10^. However, in the consideration of the heterogeneity and the complexity of structure and composition, HAs from different sources show different structures and properties^4^. Additionally, the characteristics of HAs changed with different raw material processes and extraction technologies^11^.

Lignite and weathered coal are abundant in China. Due to higher oxidation degree and lower calorific value, they are unsuitable to serve as power generation fuel or coking coal^12,13^. It was reported that global coal consumption had shown a decreasing trend in recent years. HAs are abundant and naturally occur in coal. Extracting high-added substances such as HAs from coal provided opportunities for the application of lignite and weathered coal^13–15^. However, HAs derived directly from coal often show the problems of low yield, high ash content and limited number of active functional group^16^. As two determinants, raw material selection and isolation procedure would affect the chemical character and extraction efficiency of obtained HAs from potential organic source^17^.

Coal oxidation is the most direct way to promote the yield and enrich functional groups of HAs^18^. As reported by Semenova et al.^19^, HAs could directly react with ozone in brown coal, modify the functional composition, increase the carboxyl group content. Zhumanova et al.^20^ reported that sulfuric acid promote the conversion of carbon (C) in coal to a greater extent without increasing the loss of nitrogen (N) and oxygen (O). In addition, by applying hydrogen peroxide (H_2_O_2_), potassium permanganate (KMnO_4_) and nitric acid (HNO_3_), coal oxidation can change the carbon chain structure of HAs and increase the number of active functional groups, such as hydroxyl and carboxyl^21–23^.

H_2_O_2_ oxidation increase the solubility of macromolecular structure in coal^24^. Meanwhile, large number of H and O atoms are introduced into coal, which can increase the richness of functional group^25,26^. Recently, some studies have shown that the properties of HAs change and have promoting effect on corn growth after the oxidation by H_2_O_2_^27^. In practical, coal oxidation or HAs processed by H_2_O_2_ both show excellent performance. However, ultimate purpose of these studies mainly focuses on the production of low molecular weight organic substances, with an obvious example of methane and carboxylic acid. Until now, few studies have paid attention to the research on the yield, composition, functional group and utilization of the extracted HAs in the coal oxidized by H_2_O_2_.

In this study, five coal samples were selected from different regions of China. They were oxidated by H_2_O_2_ and HAs were extracted from original coals and its oxidizition samples. The purposes of the study were to: (1) investigate the characterization of HAs from original coal and oxidized coal and compare their differences in yield, chemical characteristic and spectral property, and (2) assess the value of coal oxidation with H_2_O_2_ for HAs production and develop related nitrogen adsorption technologies.

## Results

### Yield of HAs

**Figure 1:**
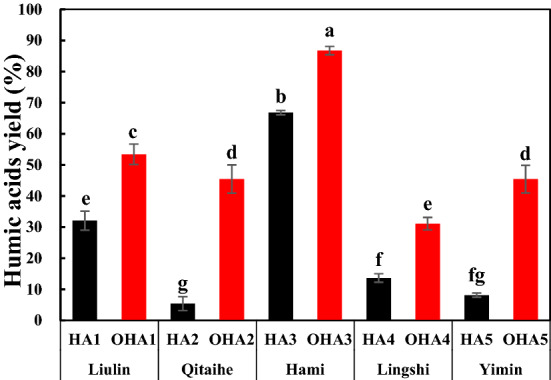
Yields of HAs. HA1–HA5, the HA extracted from original coal (black); OHA1–OHA5, the HAs extracted from oxidized coal (red); Liulin, Qitaihe, Hami, Lingshi and Yimin, the origin of coal.

HAs yield depended on extraction and refinement rather than the proportion of HAs in coal. The yields of the HAs extracted from original coal and oxidized coal were presented in Fig. 1. The yield of the HAs extracted from original coal accounted for 5.42% to 66.79% of the coal weight, and the lowest and highest yield were obtained in HA2 and HA3. Compared to that of the HAs extracted from original coal, the yield of the HAs extracted from the oxidized coal significantly increased. After oxidation, the yields of OHA1-OHA5 were ranged from 31.11% to 86.74%, HAs yield of the oxidized coal compared with those of the original coal increased from 17.47% to 40.03%.

### Ash and elemental composition of HAs

The ash contents of HAs were shown in Table 1. HA2 had the highest ash content of 18.5%, 6.15% larger than that of OHA2. In addition, the contents of ash in other HAs samples ranged from 0.50% to 2.66%. The ash contents of OHA1, OHA3, OHA4 and OHA5 were all higher than those of the HAs derived from original coal. This indicated that the ash content of HAs depended on both coal origin and sample preparation procedure. In general, coal oxidization with H_2_O_2_ could increase the ash content of HAs.

**Table 1:** Ash content, elemental composition and atomic ratio of HAs.

Samples		Ash (%)	C (%)	H (%)	N (%)	O (%)	S (%)	Atomic ratio		
								O/C	H/C	O/H
Liulin^a^	HA1^b^	0.82	58.43	4.01	1.51	31.6	1.45	0.41	0.82	0.49
	OHA1^c^	1.87	55.81	3.47	1.51	31.98	1	0.43	0.75	0.58
Qitaihe	HA2	18.62	58.94	4.1	1.28	30.11	0.6	0.38	0.83	0.46
	OHA2	12.47	45.28	3.22	0.97	30.95	0.34	0.51	0.85	0.6
Hami	HA3	1.53	60.12	3.68	1.34	32.08	0.17	0.4	0.74	0.54
	OHA3	1.61	56.84	3.79	1.32	33.76	0.13	0.45	0.8	0.56
Lingshi	HA4	0.91	58.68	3.9	1.67	32.24	0.96	0.41	0.8	0.52
	OHA4	1.6	57.41	3.75	1.66	33.3	0.92	0.44	0.78	0.56
Yimin	HA5	0.5	61.84	4.02	1.02	30.78	0.17	0.37	0.78	0.48
	OHA5	2.66	57.34	4.03	1.02	32.65	0.14	0.43	0.84	0.51

^a^The location of coal.

^b^The HAs extracted from original coal.

^c^The HAs extracted from oxidized coal.

Elemental composition values were shown in Table 1 in the form of atomic percent. HA1-HA5 were comparable in elemental composition, and their contents of C, H, N, O, and S were ranged from 58.43% to 61.84%, 3.68% to 4.10%, 1.02% to 1.67%, 30.11% to 32.24%, and 0.17% to 1.45%, respectively. Especially, HA5 performed the highest C content and H content but lowest in O content, which indicated that the HAs from original lignite had the lowest content of oxygen-containing groups. The ratios of O/C, H/C and O/H of HA1–HA5 were around 0.4, 0.8 and 0.5, showed similar elemental composition between samples.

Coal samples oxidized by H_2_O_2_ appeared changes in the elemental composition of HAs (Table 1). Among them, C and S contents of OHA1-OHA5 were lower than those of HAs derived from original coal, while O content showed an opposite trend compared with C and S content. H content exhibited irregular variation, and N content had slight variation. O/C ratio and O/H ratio increased, but H/C ratio performed irregular change. O/C ratio reflected the amount of oxygen-containing group, suggesting that coal oxidized by H_2_O_2_ increased the amount of the oxygen-containing group of HAs.

### E4/E6 ratio and the acidic functional group content of HAs

**Figure 2:**
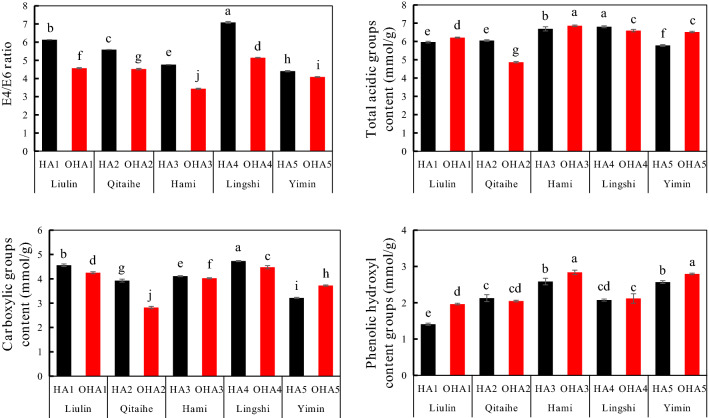
E4/E6 ratios and acidic functional group contents of HAs. HA1–HA5, the HAs extracted from original coal (black); OHA1–OHA5, the HAs extracted from oxidized coal (red); Liulin, Qitaihe, Hami, Lingshi and Yimin, the origin of coal.

E4/E6 ratio was often used to estimate the molecular weight and aromaticity of HAs^28,29^. E4/E6 ratios and acidic functional groups were shown in Fig. 2. E4/E6 ratios of the extracted HAs from original coals were ranged from 4.40 to 7.09, and significantly decreased (ranged from 7.27 to 27.73%) after oxidation.

As dominant oxygen-containing functional groups of HAs, acid functional groups (including carboxyl group and phenolic hydroxyl group) were regarded as vital indicators of HAs^30^. As shown in Fig. 2, contents of total acidic groups in HA1–HA5 ranged from 5.78 to 6.80 mmol g^−1^, with the highest in HA4 and the lowest in HA3. Acidic groups in the HAs extracted from oxidized coal significantly changed. The contents of the total acidic group in OHA2 and OHA4 increased by 19.61% and 3.09% by contrast with those of the original coal. However, the contents of the total acidic group in OHA1, OHA3 and OHA5 decreased by 4.1%, 2.51%, and 12.69% compared with those of the original coal.

Carboxyl group in HA1-HA5 were ranged from 3.21 to 4.73 mmol g^−1^ in content, and account for 55.53% to 76.42% of the total acidic group. By contrast with HAs from original coal, the content of carboxyl group decreased by 2.1% to 28.15% in OHA1-OHA4, but increased by 15.80% in OHA5. These results showed that the carboxyl group content of HAs extracted from weathered coal decreased after the oxidation by H_2_O_2_, but increased from lignite.

The contents of phenolic hydroxyl group in HA1-HA5 ranged from 1.4 to 2.58 mmol g^−1^. Compared to the HAs extracted from original coal, the contents of phenolic hydroxyl group in OHA1, OHA3, OHA4 and OHA5 increased by 55.56%, 25.4%, 4.39% and 22.6%, whereas decreased by 8.23% in OHA2.

### Main structure of the HAs revealed by FTIR spectra

**Figure 3:**
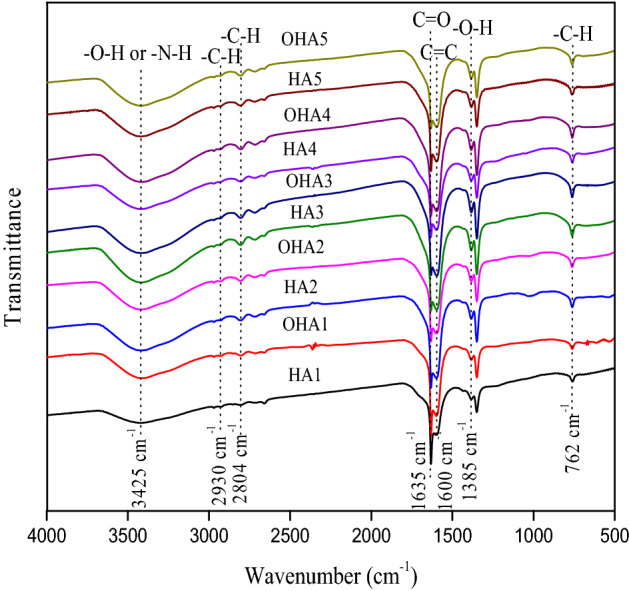
FTIR spectraof HAs. HA1–HA5, the HAs extracted from original coal; OHA1–OHA5, the HAs extracted from oxidized coal.

FTIR spectra could provide information to prove the existence and types of HAs bonds^13^. FTIR spectra of HAs were shown in Fig. 3. In general, spectra of all HAs samples were similar in primary absorption bands. Based on previous studies^13,17,31^, broad peak at approximately 3425 cm^−1^ was a sign of the N–H orhydroxy (OH) stretching vibration. Absorbance peaks at 2930 cm^−1^ and 2804 cm^−1^ were attributed to the C–H stretching vibration of aliphatic CH_3_ and CH_2_. Absorbance peak at 1635 cm^−1^ was the C=O stretching vibration of carboxylic acid. Moreover, strong absorbance peak at 1600 cm^−1^ was caused by the C=C stretching vibration of aromatic ring, which demonstrated that aromatic ring was an main structure of HAs. Absorbance peak at 1385 cm^−1^ was brought by OH deformation and C–O stretching vibration of phenolic hydroxy. Meanwhile, absorbance peak at 762 cm^−1^ represented the stretching vibration of aromatic C–H. Relative transmittance intensities of the HAs revealed by FTIR spectra were tabulated in Table 2, showed difference between locations and peaks. Relative transmittance intensity of HAs extracted from original coals were higher than those of oxidized coals at peak of 3425 cm^−1^ except Liulin location, lower at peaks of 2930 cm^−1^ and 2804 cm^−1^ except Hami location, higher at peaks of 1635 cm^−1^ and 1600 cm^−1^ except Hami location. With the decreasing of peak, variances were greater between treatments.

**Table 2:** Relative transmittance intensity of HAs revealed by FTIR spectra.

Sample		Relative transmittance intensity (%)						
		3425 cm^−1^	2930 cm^−1^	2804 cm^−1^	1635 cm^−1^	1600 cm^−1^	1385 cm^−1^	762 cm^−1^
Liulin^a^	HA1^b^	12.69	15.52	15.85	8.20	10.75	16.90	20.07
	OHA1^c^	13.88	16.75	16.81	9.31	9.25	16.29	17.70
Qitaihe	HA2	12.04	17.41	17.41	8.25	7.43	17.69	19.77
	OHA2	11.75	17.62	17.74	7.68	6.97	17.62	20.62
Hami	HA3	10.72	18.99	18.97	7.40	5.15	17.80	20.97
	OHA3	10.00	17.68	17.74	7.91	5.37	18.98	22.32
Lingshi	HA4	11.43	17.02	17.12	8.51	7.54	17.27	21.12
	OHA4	11.56	18.22	18.19	7.44	6.04	18.04	20.51
Yimin	HA5	12.12	17.14	17.24	9.09	8.32	16.99	19.10
	OHA5	10.80	18.38	18.45	7.70	5.75	17.66	21.25

^a^The location of coal.

^b^The HAs extracted from original coal.

^c^The HAs extracted from oxidized coal.

### Primary carbon structure of the HAs revealed by ^13^C NMR spectra

**Figure 4:**
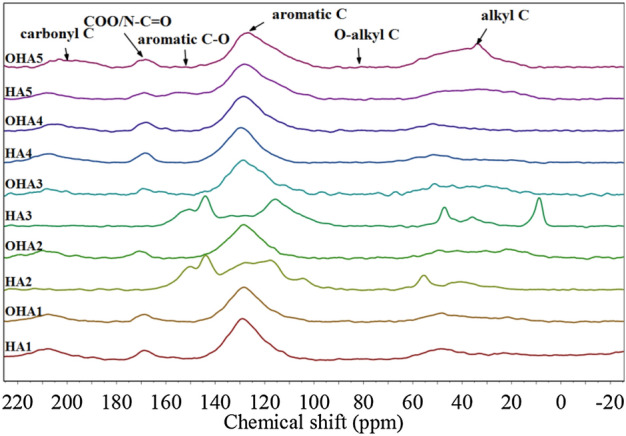
^13^C NMR spectraof HAs. HA1–HA5, the HAs extracted from original coal; OHA1–OHA5, the HAs extracted from oxidized coal.

To further analyze the structural characteristics of HAs, all HAs samples were characterized by ^13^C NMR spectroscopy, which could clarify the detailed distribution of the carbon functional group of HAs^32^. HAs spectra was showed in Fig. 4. Carbon in chemical shift range of 0–60 ppm represent edalkyl C. Weak peak in the chemical shift region of 60–98 ppm meant O-alkyl C. The highest abundance in all spectra occurred in the chemical shift region of 98–145 ppm, indicating that aromatic C was the main carbon structure in HAs. Carbons in the chemical shift region of 145–160 ppm and 160–185 ppm represented aromatic C–O and COO/N–C=O, respectively. Moreover, the peak within 185–220 ppm represented carbonyl C. Obvious differences were observed between HAs from oxidized coal and original coal. For example, there was a quite high peak of HA3 in the chemical shift region of 0–20 ppm, while OHA3 did not appear.

The relative distribution of carbon functional groups were quantified by integrating ^13^C NMR spectra, as shown in Table 3. HAs extracted from original coal showed differences in carbon functional group. The proportion of aromatics C in HA1-HA5 were ranged from 49.8% to 64.94%, followed by byalkyl C which accountting for 15.19% to 27.07%. O-alkyl C, aromatic C–O, COO/N–C=O and Carbonyl C accounted for 0.34% to 2.22%, 0.51% to 17.2%, 0.25% to 9.87% and 0.33% to 14.91% in carbon, respectively. Abundance of carbon functional group HAs from oxidized coal were also changed. Compared with the HAs from original coal, the proportion of aromatic C in OHA1, OHA3 and OHA4 increased by 12.03%, 3.82% and 9.81%, whereas decreased by 10.42% and 8.61% in OHA2 and OHA5, The proportion of alkyl C in OHA1 and OHA4 decreased by 5.14% and 4.49%, and increased by 14.3%, 2.76% and 11.6% in OHA2, OHA3 and OHA5, respectively. In addition, there was also an inconsistent change in COO/N–C=O. The proportion of Aromatic C-O in OHA4 was 0.05% higher than that of HA4, and the proportion of Carbonyl C in OHA4 was 3.78%, lower than that of HA4. However, by contrast with the extracted HA from original coal, the proportions of Aromatic C-O and Carbonyl C in OHA1, OHA2, OHA3 and OHA5 were opposite to that of OHA4. Moreover, OHA1, OHA2, OHA4 and OHA5 had lower proportions of O-alkyl C under the comparison with that of original coal.

**Table 3:** The ^13^C NMR spectral integral of HAs.

Samples		Assignment at different chemical shift regions (ppm) and relative proportions (%)					
		0–60	60–98	98–145	145–160	160–185	185–220
		Alkyl C	O-alkyl C	Aromatic C	Aromatic C–O	COO/N–C=O	Carbonyl C
Liulin^a^	HA1^b^	27.07	1.05	49.80	8.72	9.87	3.49
	OHA1^c^	21.93	0.11	61.83	0.33	5.45	10.35
Qitaihe	HA2	15.19	1.67	64.94	17.2	0.67	0.33
	OHA2	29.49	0.35	54.52	0.55	4.65	10.44
Hami	HA3	25.48	1.17	56.04	16.74	0.25	0.33
	OHA3	28.24	1.98	59.86	0.42	4.00	5.51
Lingshi	HA4	17.87	2.22	57.57	0.51	6.91	14.91
	OHA4	13.38	0.63	67.38	0.56	6.94	11.13
Yimin	HA5	25.69	0.34	56.99	6.34	4.43	6.21
	OHA5	37.29	0.24	48.38	0.33	4.19	9.57

^a^The location of coal.

^b^The HAs extracted from original coal.

^c^The HAs extracted from oxidized coal.

### Otherness among the presented HAs by principal component analysis

**Figure 5:**
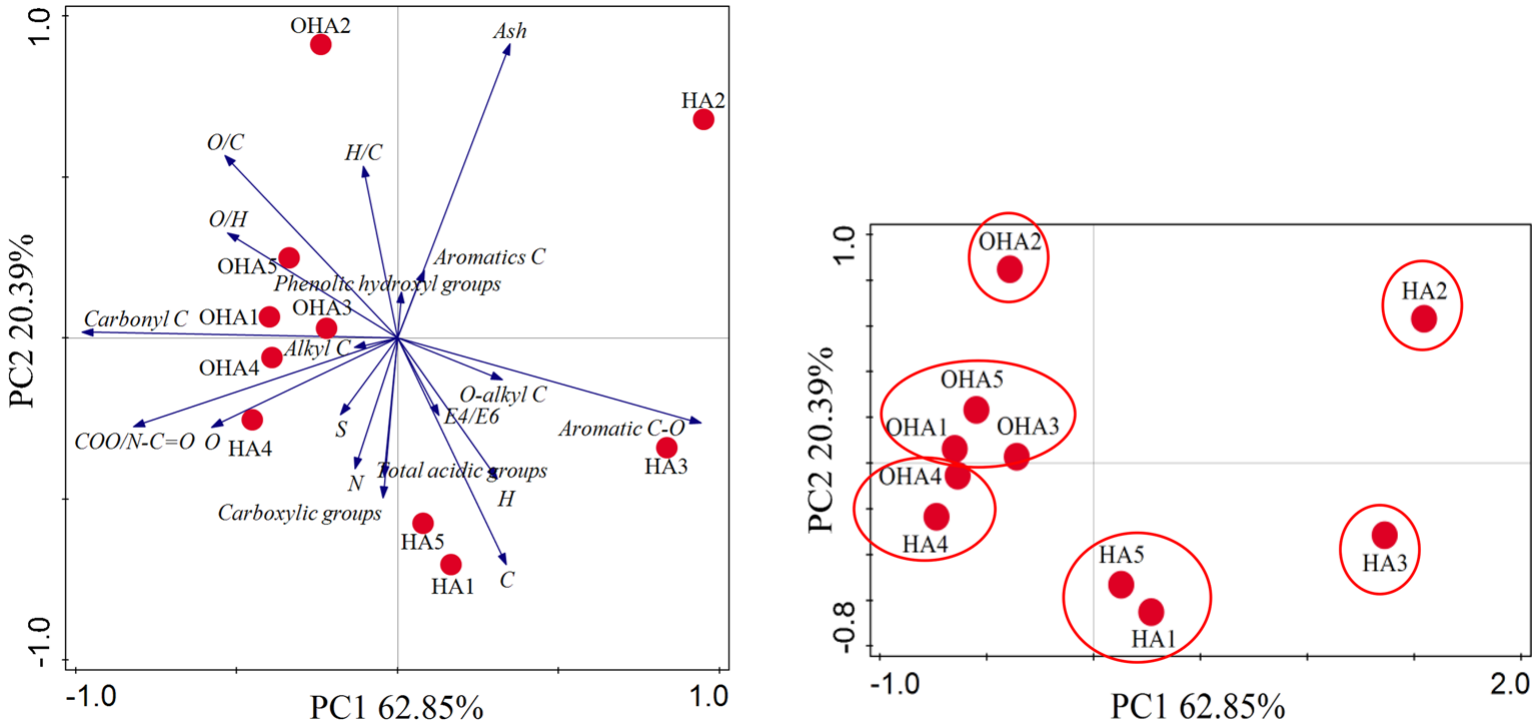
Principal components analysis (PCA) for the HAs data from the acidic functional groups contents, the ratios of atomic and E4/E6, and the relative number of carbon types. HA1–HA5, the HAs extracted from original coal; OHA1–OHA5, the HA extracted from oxidized coal.

Principal component analysis (PCA) was used for the analysis of HAs data to obtain more information^33^. According to PCA results in Fig. 5, 83.24% total variance was explained on basis of the content of acidic functional groups, atomic ratio, E4/E6 ratio and the relative number of carbon types for each HAs sample. OHA2, HA2 and HA3 were independent of each other because of their unique predominance. H/C ratio and ash content were higher in OHA2, whereas aromatic C was predominant in HA2 and aromatic C–O and O-alkyl C were predominant in HA3. HA5 and HA1 clustered in PC1 (62.82%) because of the predominant contents of C and H and E4/E6 ratio. OHA1, OHA3 and OHA5 clustered in PC2 (20.39%) because of the predominant ratios of O/H and O/C and the relative number of Carbonyl C. HA4 and OHA4 clustered in negative values because of the predominant content of C and the relative numbers of COO/N–C=O and Alkyl C. It could be clearly seen that HA1–HA4 were far away from each other, which indicated that there was large property difference among those HAs from the original coal. In addition to HA4 and OHA4, distances were far apart between HA1 and OH1, HA2 and OH2, HA3 and OHA3, and HA5 and OHA5, respectively. It indicated that properties of the extracted HAs from oxidized coal and original coal were significantly different.

### Relationship among ammonium nitrogen adsorption, acidic group content and E4/E6 of HAs

**Figure 6:**
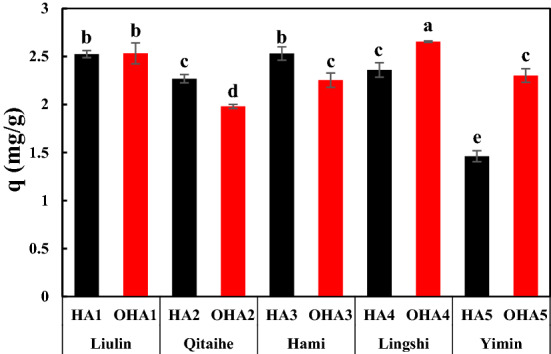
Nitrogen adsorption of HAs, HA1–HA5, the HAs extracted from original coal (black); OHA1–OHA5, the HAs extracted from oxidized coal (red); Liulin, Qitaihe, Hami, Lingshi and Yimin, the origin of coal.

HAs usually have perfect adsorption capacity, so they were considered as nitrogen fertilizer adsorbent to promote the efficient utilization of nitrogen fertilizer^31,34^. Different HAs samples had differed nitrogen adsorption capacity (Fig. 6). The nitrogen adsorption capacity of the HAs sample ranged from 1.46 to 2.65 mg N g^−1^, with the lowest in HA5 and the highest in OHA4. There was no significant difference between HA1 and OHA1 in nitrogen adsorption capacity. In addition, nitrogen adsorption capacity also showed significantly different between the extracted HAs from oxidized coal and original coal. Nitrogen adsorption capacity in OHA2 and OHA3 were lower than those of HA2 and HA3. In contrast, the nitrogen adsorption capacity of OHA4 and OHA5 were higher than those of HA4 and HA5. As seen from Table 4, there was a significantly positive correlation between the content of carboxyl group and nitrogen adsorption. It indicated that carboxyl group played a major role in the adsorption process of nitrogen.

**Table 4:** Correlation between nitrogen (N) adsorption and different acidic functional groups.

	Total acidic group	Carboxylic group	Phenolic hydroxyl group	E4/E6
N adsorption	0.522	0.774**	− 0.333	0.294

**Significant correlation at 0.01 level.

## Discussion

### Yield of the HAs

This study demonstrated that there was great difference in yield of HAs extracted from oxidized coal. The difference lied in locations of coal^4^. The highest yield of HA3 of original coal from Hami was due to lower ash content (16.38%) and higher HAs content, whereas it was on the contrary with the coal from Qitaihe. However, the main hindrance to use HAs in large scale relies to extraction of HAs from coal^18^. Therefore, pollution-free and low-cost technologies were particularly important to promote the yield of HAs. After the oxidation of coal by H_2_O_2_, the yield of HAs increased from 17.47% to 40.03% significantly. Similar to the oxidation process of aerosphere, the mild oxidation of H_2_O_2_ could degrade some high-molecular-weight constituents of organic matter in coal, and increase the number of oxygen-containing functional groups, thus increasing the solubility of organic matter and leading to greater HAs production^35,36^. It was observed that the reaction of extracting HAs by H_2_O_2_ was more strongly. In previous studies, HAs were usually extracted from dried coal, while alkali solution treatment was directly carried out after H_2_O_2_ oxidation in this study and resulted in an increase of HAs yield. The reason might be that HAs dissolving in coal, released trace-amount transitional metals to catalyze the residual H_2_O_2_ to produce reactive radical species^37^, which further promoted the degradation of organic compound.

### The characteristics of the HAs

H_2_O_2_ was able to effectively solubilize the macromolecular structure of low rank coal and promote the production of low molecular substance^38^. In this study, oxidized by H_2_O_2_ increased the yield of HAs, which meant that less organic matter in coal was converted to low molecular weight matter. In accordance with the result proposed by Doskočil et al.^36^ who found that the decrease in temperature and time of the reaction between H_2_O_2_ and lignite resulted in decrease in the yield of hydrophilic fraction and the mass loss of coal. These oxidative solubilization effects, such as oxidizing weak –C–O– linkage in coal, produced CO_2_ and water-soluble organic compounds^25^, changed the structure of organic matter, and thus modified the extraction process of HAs. This study also found some evidences to prove this view, such as the decrease of C content and relative aromatic C-O content of the extracted HAs from oxidized coal. Compared with the extracted HAs from original coal, the HAs from oxidized coal had lower content of carboxylic group and higher content of phenolic hydroxyl. This could be explained by the study of Miura et al.^25^, Mae et al.^38^ and Novikova et al.^39^, who reported that HO radical of H_2_O_2_ promoted the formation of large amount of carboxyl group, phenolic hydroxyl and other oxygen functional groups. Then, the aromatic ring with carboxyl group was decomposed to produce small molecule acids, causing the decrease of carboxylic group in HAs. The trend of carboxyl group in lignite was opposite to the weathered coal, which was possibly due to less relative aromatic ring structure in lignite, while carboxylic group was mostly formed on aliphatic chain.

Large variation in E4/E6 ratio (4.40 to 7.09) of the extracted HAs from original coal indicated that their aromaticity, molecular weight and condensation were greatly different^3,13^. Generally, the E4/E6 ratio decreases with increasing aromatic condensation and molecular mass^28,29^. A lower oxygen content causes decreases in ratio^40^. In this study, the extracted HAs from oxidized coal decreased in E4/E6 ratio compared with the extracted HA from original coal. However, the content of oxygen increased and the proportion of aromatic C decreased, suggesting that the extracted HAs from oxidized coal had higher molecular weight but poor aromaticity.

### Potential utilization of HAs

Adsorption and complexation of HAs depended on various active functional groups^34^. In this study, it was proved that carboxyl group was positively correlated with NH_4_^+^–N adsorption, which is in agreement with the finding of Chassapis et al.^6^ who revealed the coordination of metal ions with carboxyl and phenolic groups of oxygen-rich humic substances by UV–Vis and TR spectra. Based on the NH_4_^+^–N adsorption capacity of HAs, H_2_O_2_ oxidation was beneficial to coals from Yimin lignite, but the oxidation was unconspicuous for the Qitaihe coal, with the increase and decrease of the carboxyl group content of the extracted HAs to about 28.15% and 15.80%, respectively. As far as we know, one way to reduce the loss of NH_4_^+^–N in agriculture was colloid adsorption. Thus, this study provided raw material for the efficient utilization of NH_4_^+^–N fertilizer. Moreover, higher oxygen and oxygen/carbon contents of HAs promoted the root growth of maize^27^. Rich carboxyl and phenolic groups in HAs allowed deprotonation and promoted plant growth and nutrition uptake^41^. Moreover, HAs can stabilize amorphous calcium carbonate and delay its transformation to a stable state. In this process, the carboxyl group of HAs plays a key role^42^. These results provided promising prospects for the use of the extracted HAs from oxidized coal in agriculture.

## Conclusion

The extraction of HAs products from coal has been regarded as an effective way to coal utilization. In this study, coal oxidized by H_2_O_2_ promoted the yield of HAs, and modified their structure and composition. In general, the manifestation of this modification referred to the reduction of C, carboxyl group, the ratio of E4/E6, O-alkyl C and Aromatic C–O, the increase of ash, O and phenolic hydroxyl group, the ratios of O/C and O/H, and Carbonyl C. Moreover, it had been proved in this study that carboxyl group was the main functional group of NH_4_^+^–N adsorption, which provided the application prospect of carboxy-rich HAs in the efficient utilization of NH_4_^+^–N fertilizer in agriculture.

## Material and method

### Material

**Figure 7:**
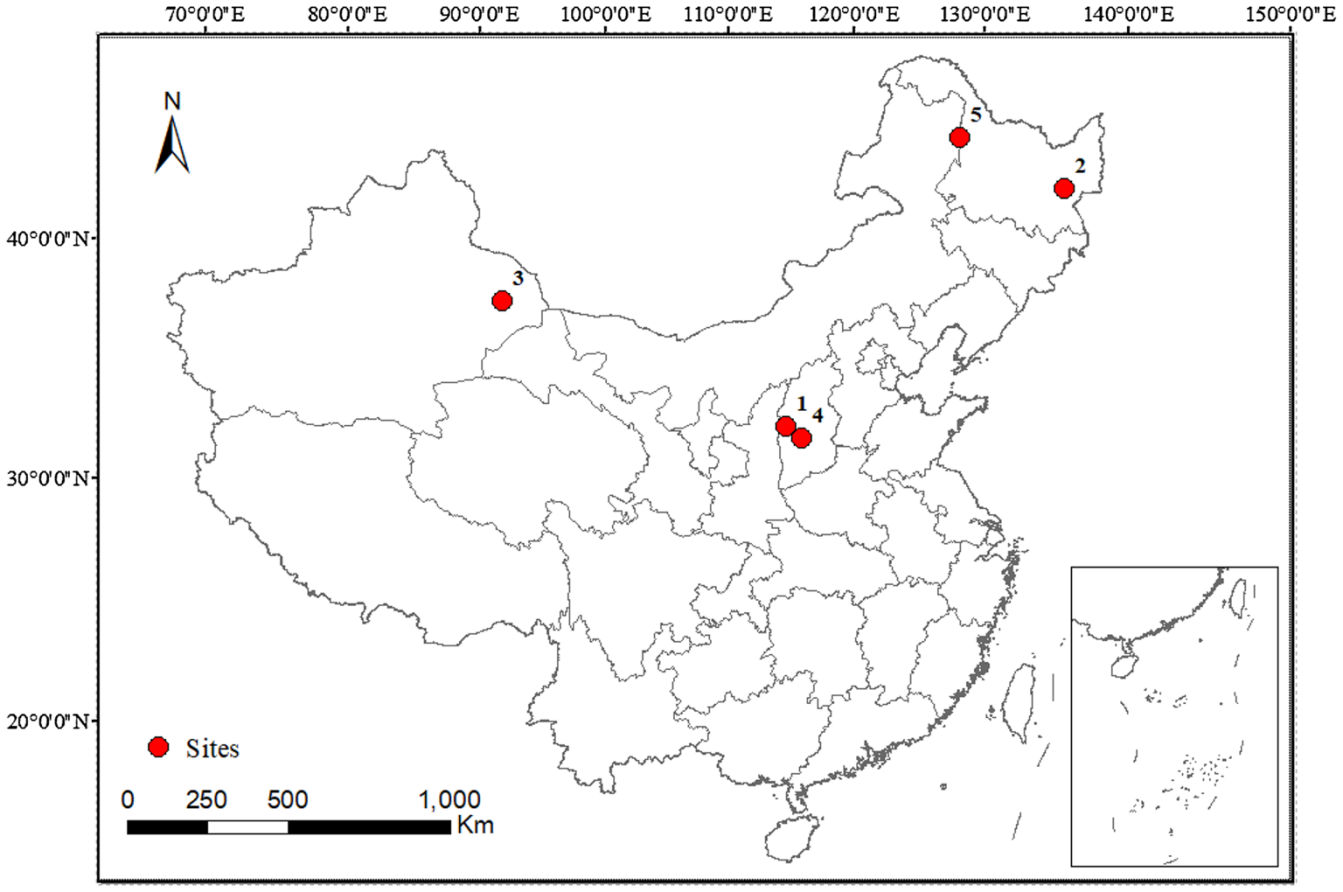
Sites location of the five collected coals, 1, Liulin; 2, Qitaihe; 3, Hami; 4, Lingshi; 5, Yimin.

Five coals were selected from main coal-producing areas of China (Fig. 7). Among them, four kinds of weathered coal were collected from Hami City (42°49′N, 93°31′E, in Xinjiang Uygur Autonomous Region, northwest of China), Qitaihe City (45°45′N, 130°51′E, in Heilongjiang Province, northeast of China), Liulin County (37°27′N, 110°54′E, in Lvliang city, Shanxi Province, north of China), and Lingshi County (36°50′N, 111°46′E, in Jinzhong city, Shanxi Province, north of China), respectively. One kind of lignite was collected from Yimin Sumu (49°26′N, 124°57′E, in Hulunbuir city, Inner Mongolia Autonomous Region, north of China). Ash contents of the coal were 24.99% from Liulin, 61.52% from Qitaihe, 16.38% from Hami, 35.56% from Lingshi and 34.21% from Yimin. All coal samples were air-dried, ground and sieved with an 80-mesh sieve for subsequent utilization.

### Samples oxidized by H_2_O_2_

The coal sample was oxidized by 10% H_2_O_2_ (solid to liquid ratio equal to 1:5). The mixture was fully stirred and oxidized for 6 h (control temperature ≤ 60 °C).

### HAs extraction

The coal sample and 1 mol L^−1^ NaOH (solid to liquid ratio equal to 1:10) were mixed thoroughly by stirring and oxidizing for 12 h (control temperature ≤ 60 °C) to dissolve and generate HAs and other insoluble substances, followed by centrifugation (3500 rpm, 15 min) to remove solid residues. pH of remained solution was adjusted to less than 2 with 1 mol L^−1^ HCl, and corresponding supernatant was removed by centrifugation after 24 h standing. Next, it was soaked for 3 times by the solution (50 mL) mixing 0.1 mol L^−1^ HCl and 0.3 mol L^−1^ HF to reduce ash and other ions. For every soak, it lasted for 12 h, and the supernatant was removed by centrifugation, cleaned with deionized water until chloride-free state, dried to constant weight at 60 °C and lastly weighted. A similar method was used for HAs extraction from oxidized coal, and their difference was only that the concentration of NaOH was doubled and solution amount was halved. Finally, HAs yield was the ratio of the amount of the extracted HAs to the added coal. The five regions (Liulin, Qitaihe, Hami, Lingshi and Yimin) were named as 1 to 5. The samples extracted from original coal were labelled as HA1–HA5, and those extracted from oxidized coal were labelled as OHA1–OHA5.

### Characterization of humic acids

#### Ash and elemental composition

The content of ash was determined through muffle furnace combustion (700 °C for 4 h), and the contents of C, H, O, N and S in HA were analyzed by Vario Micro Cube Elementar (Vario EL Cube Germany).

#### E4/E6 and functional group

E4/E6 was determined by UV/VIS spectrophotometer (Analytik Jena, Germany) at 446 nm and 665 nm for HAs dissolving in 0.05 mol L^−1^ NaHCO_3_ solution (pH 8.3) at HAs concentration of 40 mg L^−1^. The content of the acidic functional group was regarded as the sum of the contents of phenolic hydroxyl group and carboxyl group. According to the methodproposed by Zhang et al.^13^, the contents of the total acidic group and carboxyl group were determined by Ba(OH)_2_ titration and Ca(CH_3_COO)_2_ titration. Total of 0.2 g of HAs was accurately weighed and placed into a 25 mL centrifuge tube, and alid was immediately plugged. Next, operations were as the following: opening the lid, accurately adding 2 mL 0.1 mol L^−1^ Ba(OH)_2_ solution, sealing the lid, and placing the container at room temperature for 48 h. The tube was shaken every two hours for 5 min during the period. After 48 h, the solution in the centrifuge tube was rapidly filtered into a 50 mL centrifuge tube, and 10 mL added into the prepared triangular flask with 10 mL 0.1 mol L^−1^ HCl solution. Three drops of phenolphthalein were added into 0.1 mol L^−1^ NaOH standard solution until the solution turned from colourless to slightly red. The content of carboxyl group was determined by reference to the determination of the total acidic group. Then, 25 mL 0.1 mol L^−1^ Ca(CH_3_COO)_2_ was substituted for 25 mL 0.1 mol L^−1^ Ba(OH)_2_. The obtained filtrate was separated into 10 mL, added with 3 drops of phenolphthalide, and titrated with standard 0.1 mol L^−1^ NaOH solution until the solution changed from colourless to slightly red.

### Fourier transformation infrared spectroscopy

Dried HAs samples and KBr were ground in an agate mortar at 1:1000, then subjected to a 6700 FTIR (TIANJINGANGDONG, China) with a smart miracle Si crystal to attenuate total reflection fitting, and lastly tested by a deuterated triglycine sulfate potassium bromide detector. The infrared spectrometer measured at a wave number of 400–4000 cm^−1^ (wavelength of 2.5–15.9 μm). The total average number of scanning per spectrum was 64 with resolution at 4 cm^−1^. The spectra were drawn with Origin 8.0.

### ^13^C NMR spectra

JNW-ECZ600R NMR spectrometer (JNM-ECZ600R, Japan) was used to identify the HAs functional group by using Solid-state ^13^C cross-polarization (CP) magic-angle spinning (MAS) and high-power dipole decoupling (DD) or applying a single 90-degree pulse excitation (SPE) with high-power decoupling. After the samples were dissolved in 0.1 mol L^−1^ NaOH, specific parameters were determined as follows: temperature at 298.15 K, rotor diameter of 5 mm, scanning number of 12,000, scanning time of 5.7 μs (90°), excitation pulse collection time of 0.43 s, spectra width of 37538 Hz, pulse delay of 3 s, and gated decoupling for 1H. Besides, free induction attenuation signal was widened by 25 Hz line, and chemical displacement was denoted by DSS (2,2-dimethyl-2-silapentane-5-sulfonate) signal. Mestrenova 9.0.1 was used to draw, smooth and integrate spectra.

### Adsorption experiment

Adsorption experiment was conducted to compare the adsorption effect of different HAs on ammonium nitrogen (NH_4_^+^–N). The experiment was performed in a set of 50 mL polypropylene plastic tubes with 0.5 g HAs solid, 30 mL of 100 mg N L^−1^ ammonium sulfate (pH 5.0), and 1 drop of toluene to inhibit microbial growth. The control group did not contain HAs, and all treatments were repeated for three times. All tubes were capped and placed in an incubator shaker (SHZ-82A) (180 rpm, 24 h) at 25 °C. Then, they were centrifuged for 10 min at 4000 rpm for the separation of HAs solid and solution. Lastly, the supernatant was used to determine the concentration of N with the help of a flow analyzer.

### Statistical analysis

One-way analysis of variance (ANOVA) with Duncan’s test was utilized to evaluate significant differences (P < 0.05) in the yield, E4/E6 ratio, acidic functional group content and N adsorption content of HAs through the statistical analysis software SPSS (Version 22). Canoco software (version 4.5) was applied for principal component analysis (PCA) of elemental composition, atomic ratio, acidic functional group, E4/E6 ratio and NMR integral data. Pearson correlation analysis was conducted for N adsorption content and acidic functional groups by using SPSS software (Version 22).
